# Supplementary material for: Stoichiometric 14-3-3ζ binding promotes phospho-Tau microtubule dissociation and reduces aggregation and condensation
Source: Commun Biol. 2025 Jul 31;8:1139. doi: 10.1038/s42003-025-08548-0 (PMC12313985; doi:10.1038/s42003-025-08548-0)
Supplement: Supplementary file 1 — Supplemental Figures [file 42003_2025_8548_MOESM1_ESM.docx]

**Supplemental** **Material** (Supplemental Figures S1-S5)

**Supplemental** **Figure S1. Tau phosphorylation and TaupS214/pS324 and pS2 peptide binding to 14-3-3ζ.**

**a,** Western blots of cell lysates from cultured primary mouse neurons show phosphorylation of endogenous mouse Tau at S214 and S324. **b,** Full Western blot membranes for Figure 2d. **c,** Western blots of recombinant Tau modified by kinases in vitro and probed for phosphorylation sites not relevant for 14-3-3 binding **d,** Amino acid sequences (letter code) of Tau phospho-peptides. Phospho-sites are marked in red letters, linker region in blue letters. **e,** Independent experimental replicates of fluorescence anisotropy measurements for FITC-labeled Tau peptides pS2, Tau_pS214_, and Tau_pS324_ (related to Fig. 2e). Data shown as mean±SD, N=3 technical replicates per experiment. **f,** Independent experimental replicates of fluorescence anisotropy measurements for full-length PKA-Tau variants competing with pS2-FITC bound to 14-3-3ζ. Data shown as mean±SD, N=3 technical replicates per experiment. **g,** Phos-Tag gel shows efficient phosphorylation of Tau by PKA and Cdk5 compared to non-phosphorylated Tau.

**Supplemental Figure S2. Western bots of MT pelleting assay and 14-3-3ζ in condensation buffer.**

a, Western blots for human Tau and 14-3-3 in four independent MT pelleting assays used for quantification in Fig. 3a. Note, Blot0 was additionally blotted against tubulin to show the enrichment of MTs in the pellet fraction, but is missing the (MT+Tau+14-3-3 sample). **b,** Representative images of different 14-3-3ζ concentrations (incl. 2% 14-3-3ζ-DyLight650) in condensation assay buffer (HEPES, pH7.4, 5% PEG). 14-3-3ζ and 14-3-3ζ^R127A^ alone do not form condensates even at 100 μM. Scale bars = 20 μm.


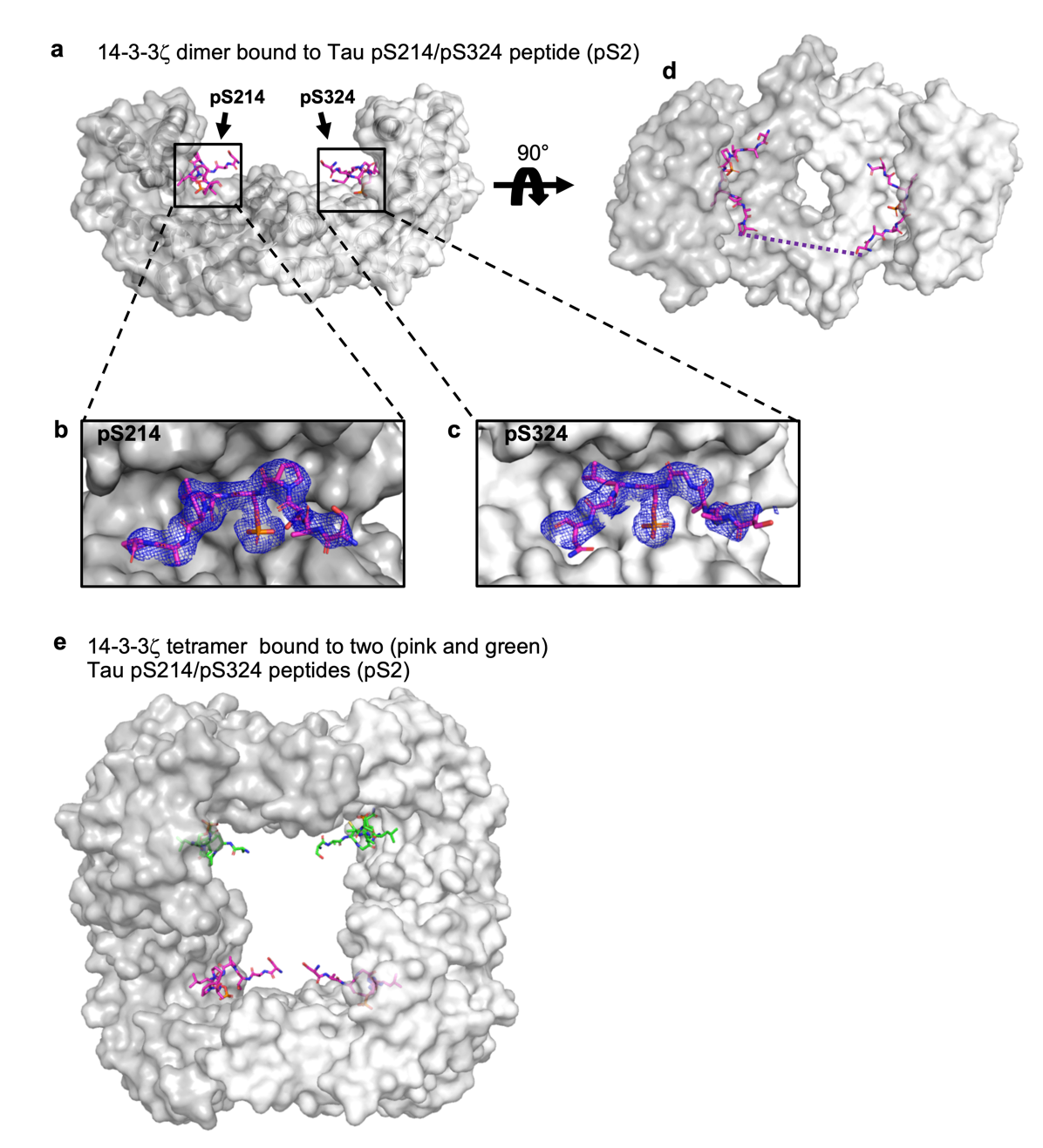


**Supplemental Figure S3.**

**a,** Second 14-3-3ζ dimer part of the asymmetric unit in complex with Tau phospho-peptide pS2. Cartoon plot with semi-transparent surface of the 14-3-3ζ dimer (gray) complexed with the pS214 and pS324 binding sites of the pS2 (pink rods). **b+c,** Close-up of the 14-3-3ζ binding groove in complex with individual pS2 binding sites (pink rods). Final 2Fo-Fc electron density map of pS2 is shown as blue mesh (contoured at 1s). **d,** Top view of 14-3-3ζ (grey semi-transparent surface) in complex with pS2 binding motifs around pS214 and pS324 (pink rods) connected by the unstructured linker (purple dotted line). **e,** Crystal packing of pS2:14-3-3ζ complexes. Two 14-3-3ζ dimers forming a tetramer (gray semi-transparent surfaces) are bound to two two pS2 peptides (green and pink rods).


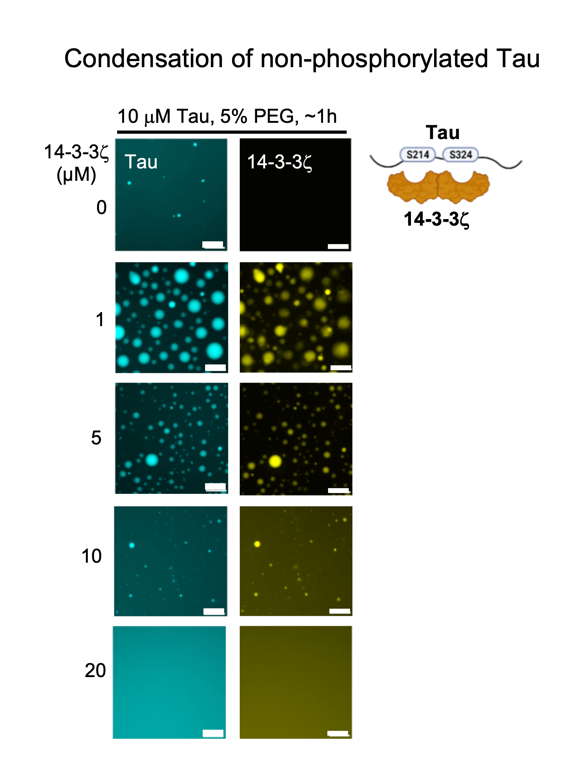


**Supplemental Figure S4. 14-3-3ζ induces Tau condensation at net charge matching.**

Representative images of 10 μM non-phosphorylated Tau with increasing 14-3-3ζ concentrations (0, 1, 5, 10, 20 μM in 25 mM HEPES, pH 7.4, 1 mM DTT, 5% (w/v) PEG; 2% Tau-DyLight488, 2% 14-3-3ζ-DyLight647). Scale bars = 20 μm.

**Supplemental Figure S5. Tau_pS214/pS324_ in cell lysates.**

**a,** Western blots of cell lysates from mouse cortical primary neurons confirm the presence of both Tau_pS214_ and Tau_pS324_ phosphorylation in neurons. **b,** Western blots of cell lysates from human glioblastoma (SH-SY5Y), mouse neuroblastoma (Neuro2a), and human kidney (HEK293) cells show no content of Tau_pS214_ or Tau_pS324_ in either cell line. GAPDH was used as loading control.
