# Supplementary material for: Stoichiometric 14-3-3ζ binding promotes phospho-Tau microtubule dissociation and reduces aggregation and condensation
Source: Commun Biol. 2025 Jul 31;8:1139. doi: 10.1038/s42003-025-08548-0 (PMC12313985; doi:10.1038/s42003-025-08548-0)
Supplement: Supplementary file 2 — Description of Additional Supplementary Files [file 42003_2025_8548_MOESM2_ESM.docx]

Description of Additional Supplementary Files

**File name:** Supplementary Data 1

**Description:** Source data for quantitative experiments.
