# Supplementary material for: Stoichiometric 14-3-3ζ binding promotes phospho-Tau microtubule dissociation and reduces aggregation and condensation
Source: Commun Biol. 2025 Jul 31;8:1139. doi: 10.1038/s42003-025-08548-0 (PMC12313985; doi:10.1038/s42003-025-08548-0)
Supplement: Supplementary file 4 — Reporting Summary [file 42003_2025_8548_MOESM4_ESM.pdf]

nature portfolio

Corresponding author(s): susanne.wegmann@dzne.de
Luc.brunsveld@tu-eindhoven.nl

Last updated by author(s): Jun 26, 2025

Reporting Summary

Nature Portfolio wishes to improve the reproducibility of the work that we publish. This form provides structure for consistency and transparency in reporting. For further information on Nature Portfolio policies, see our Editorial Policies and the Editorial Policy Checklist.

Statistics

For all statistical analyses, confirm that the following items are present in the figure legend, table legend, main text, or Methods section.

n/a	Confirmed	
☐	☒	The exact sample size (n) for each experimental group/condition, given as a discrete number and unit of measurement
☐	☒	A statement on whether measurements were taken from distinct samples or whether the same sample was measured repeatedly
☐	☒	The statistical test(s) used AND whether they are one- or two-sided<br>*Only common tests should be described solely by name; describe more complex techniques in the Methods section.*
☒	☐	A description of all covariates tested
☒	☐	A description of any assumptions or corrections, such as tests of normality and adjustment for multiple comparisons
☐	☒	A full description of the statistical parameters including central tendency (e.g. means) or other basic estimates (e.g. regression coefficient) AND variation (e.g. standard deviation) or associated estimates of uncertainty (e.g. confidence intervals)
☒	☐	For null hypothesis testing, the test statistic (e.g. F, t, r) with confidence intervals, effect sizes, degrees of freedom and P value noted<br>*Give P values as exact values whenever suitable.*
☒	☐	For Bayesian analysis, information on the choice of priors and Markov chain Monte Carlo settings
☒	☐	For hierarchical and complex designs, identification of the appropriate level for tests and full reporting of outcomes
☒	☐	Estimates of effect sizes (e.g. Cohen's d, Pearson's r), indicating how they were calculated

Our web collection on statistics for biologists contains articles on many of the points above.

Software and code

Policy information about availability of computer code

Data collection	For 14-3-3 x-ray structure:XDS
Data analysis	Statistical data analysis and curve fitting was performed using Graphpad Prism 10. Analysis of 14-3-3 sx-ray tructure:CCP4

For manuscripts utilizing custom algorithms or software that are central to the research but not yet described in published literature, software must be made available to editors and reviewers. We strongly encourage code deposition in a community repository (e.g. GitHub). See the Nature Portfolio guidelines for submitting code & software for further information.

Data

Policy information about availability of data

All manuscripts must include a data availability statement. This statement should provide the following information, where applicable:

- Accession codes, unique identifiers, or web links for publicly available datasets
- A description of any restrictions on data availability
- For clinical datasets or third party data, please ensure that the statement adheres to our policy

All raw numeric data are available in Supplemental Table T1. The X-ray structure of 14-3-3zeta in complex with pS2 peptide is available via the PDB with IDs: 8QDV (free access).

Research involving human participants, their data, or biological material

Policy information about studies with human participants or human data. See also policy information about sex, gender (identity/presentation), and sexual orientation and race, ethnicity and racism.

Reporting on sex and gender	na
Reporting on race, ethnicity, or other socially relevant groupings	na
Population characteristics	na
Recruitment	na
Ethics oversight	na

Note that full information on the approval of the study protocol must also be provided in the manuscript.

Field-specific reporting

Please select the one below that is the best fit for your research. If you are not sure, read the appropriate sections before making your selection.

☒ Life sciences ☐ Behavioural & social sciences ☐ Ecological, evolutionary & environmental sciences

For a reference copy of the document with all sections, see nature.com/documents/nr-reporting-summary-flat.pdf

Life sciences study design

All studies must disclose on these points even when the disclosure is negative.

Sample size	at least n=2-4 independent experiments with 1 or more technical replicates, depending on experiment; experimental / technical replicates reported in figure legends
Data exclusions	no data was excluded
Replication	experiments were replicated 2-4 times
Randomization	sample collection order (well and location was randomized between experimental replicates
Blinding	imaging data was analyzed blinded whenever possible

Reporting for specific materials, systems and methods

We require information from authors about some types of materials, experimental systems and methods used in many studies. Here, indicate whether each material, system or method listed is relevant to your study. If you are not sure if a list item applies to your research, read the appropriate section before selecting a response.

Materials & experimental systems

n/a	Involved in the study	
☐	☒	Antibodies
☐	☒	Eukaryotic cell lines
☒	☐	Palaeontology and archaeology
☐	☒	Animals and other organisms
☒	☐	Clinical data
☒	☐	Dual use research of concern
☒	☐	Plants

Methods

n/a	Involved in the study	
☒	☐	ChIP-seq
☒	☐	Flow cytometry
☒	☐	MRI-based neuroimaging

Antibodies

Antibodies used	mouse anti-Tau pSer202/pSer205 (AT8), Invitrogen, MN1020; rabbit anti-TaupS214, Abcam, ab170892; rabbit anti-TaupS324, Abcam, ab109401; chicken anti-Map2, Abcam, ab92434; rabbit anti-14-3-3 Abcam, ab51129; rabbit anti-total Tau, DAKO, A0024;mouse anti-pan 14-3-3 (H-8), Santa Cruz, sc-1657; mouse anti-total human Tau, Biolegend, 835201; anti-tubulin, Sigma, T6074

	DyLight405 goat anti-mouse IgG, Invitrogen, 35501BID; AlexaFluor647 goat anti-chicken IgY, Invitrogen, A21449; AlexaFluor555 goat anti-rabbit IgG, Invitrogen, A27039
Validation	all antibodies used have been previously and extensively used in the fields of Tau and 14-3-3 research.we did not perform additional validation of the antibodies.

Eukaryotic cell lines

Policy information about cell lines and Sex and Gender in Research

Cell line source(s)	HEK 293-T (Sigma, 12022001-1VL), SH-SY5Y (Sigma, 94030304-1VL), Neuro2a (Sigma, 89121404-1VL)
Authentication	DNA sequencing
Mycoplasma contamination	tested for each cell line every 6 months
Commonly misidentified lines (See ICLAC register)	na

Animals and other research organisms

Policy information about studies involving animals; ARRIVE guidelines recommended for reporting animal research, and Sex and Gender in Research

Laboratory animals	mus musculus, C57/B6, p0 pups
Wild animals	na
Reporting on sex	mixed sex
Field-collected samples	na
Ethics oversight	All animal work was performed in accordance with the German governmental animal welfare act and guidelines, and in compliance with Charité Berlin and DZNE Berlin approved animal procedures. The sacrificing of mouse pups for primary neuron preparations was approved by the German official entities based on T-CH 0019/24. Top reduce animal numbers, all animal experiments were performed in a minimum experimental/ technical repeat (3-4 experiments with 1-3 replicates).

Note that full information on the approval of the study protocol must also be provided in the manuscript.

Plants

Seed stocks	na
Novel plant genotypes	na
Authentication	na
